# Perceived risks and benefits of cigarette smoking among Nepalese adolescents: a population-based cross-sectional study

**DOI:** 10.1186/1471-2458-13-187

**Published:** 2013-03-02

**Authors:** Umesh Raj Aryal, Max Petzold, Alexandra Krettek

**Affiliations:** 1 Department of Community Medicine, Kathmandu Medical College, Kathmandu, Nepal; 2 Nordic School of Public Health NHV, Gothenburg, Sweden; 3 Centre for Applied Biostatistics, Sahlgrenska Academy at University of Gothenburg, Gothenburg, Sweden; 4 Department of Internal Medicine and Clinical Nutrition, Institute of Medicine, Sahlgrenska Academy at University of Gothenburg, Gothenburg, Sweden

**Keywords:** Susceptibility to smoking, Physical risks, Social risks, Addiction risk, Social benefits

## Abstract

**Background:**

The perceived risks and benefits of smoking may play an important role in determining adolescents’ susceptibility to initiating smoking. Our study examined the perceived risks and benefits of smoking among adolescents who demonstrated susceptibility or non susceptibility to smoking initiation.

**Methods:**

In October–November 2011, we conducted a population-based cross-sectional study in Jhaukhel and Duwakot Villages in Nepal. Located in the mid-hills of Bhaktapur District, 13 kilometers east of Kathmandu, Jhaukhel and Duwakot represent the prototypical urbanizing villages that surround Nepal’s major urban centers, where young people have easy access to tobacco products and are influenced by advertising. Jhaukhel and Duwakot had a total population of 13,669, of which 15% were smokers. Trained enumerators used a semi-structured questionnaire to interview 352 randomly selected 14- to 16-year-old adolescents. The enumerators asked the adolescents to estimate their likelihood (0%–100%) of experiencing various smoking-related risks and benefits in a hypothetical scenario.

**Results:**

Principal component analysis extracted four perceived risk and benefit components, excluding addiction risk: (i) physical risk I (lung cancer, heart disease, wrinkles, bad colds); (ii) physical risk II (bad cough, bad breath, trouble breathing); (iii) social risk (getting into trouble, smelling like an ashtray); and (iv) social benefit (looking cool, feeling relaxed, becoming popular, and feeling grown-up). The adjusted odds ratio of susceptibility increased 1.20-fold with each increased quartile in perception of physical Risk I. Susceptibility to smoking was 0.27- and 0.90-fold less among adolescents who provided the highest estimates of physical Risk II and social risk, respectively. Similarly, susceptibility was 2.16-fold greater among adolescents who provided the highest estimates of addiction risk. Physical risk I, addiction risk, and social benefits of cigarette smoking related positively, and physical risk II and social risk related negatively, with susceptibility to smoking.

**Conclusion:**

To discourage or prevent adolescents from initiating smoking, future intervention programs should focus on communicating not only the health risks but also the social and addiction risks as well as counteract the social benefits of smoking.

## Background

Smoking and the use of other tobacco products kill 15,000 people in Nepal each year [1]. A recent study suggested that 3.41% of Nepalese adolescents between 10 and14 years of age and 16.74% between 15 and 19 years of age smoke [2]. Smoking prevalence varies among schools (2%–49%) and districts (7%–29%) [3]. Studies among school-age and college students report that most students begin smoking between 13–16 years of age and that initiation age ranges between 5–18 years [4-6]. Therefore, preventing tobacco use and smoking initiation in adolescents is a public health concern that aims to reduce many chronic degenerative diseases (e.g., cardiovascular diseases, chronic respiratory diseases, and cancer) [7]. Further, cardiovascular risk factor studies reported that the risk of acute myocardial infarction is 2.61-fold higher (95% CI: 1.99–3.44) in South Asian smokers (Nepal, Bangladesh and Sri Lanka) compared with individuals outside South Asia and population attributable risk is 43% [8].

Adolescents may incorrectly believe that cigarette smoking is less risky than other behaviors, such as alcohol consumption and drug use, and they do not understand the short-term effect and addictive nature of smoking [9-11]. Many adolescent smokers understand the risks of smoking in general terms but greatly underestimate the personal risks, largely because they believe they can quit before becoming addicted [12]. Adolescents underestimate the effects of smoking and overestimate their ability to quit before smoking affects their health [12]. A systematic review revealed that youthful optimism and self-exempting beliefs about the likelihood of addiction, health risks, and consequences of smoking associate with smoking behavior [13]. Thus, adolescents begin smoking and progress toward becoming established smokers, moving from the preparation phase to a stable level of addiction [14].

In the preparation phase, nonsmoking adolescents are cognitively vulnerable or susceptible to smoking [14,15]. Susceptibility to smoking is a good predictor of smoking initiation, as measured by acceptance of friends’ smoking and the sentiment that they would like to smoke in the future [14]. To predict the different stages of smoking behavior among adolescents, Pierce et.al. and other studies successfully measured susceptibility to smoking [14-16]. Several factors associate with susceptibility, including people’s knowledge, attitudes, and perceptions about cigarette smoking [17]. Different health behavior theories have explained that psychosocial risks and protective factors, including beliefs about the risks and perceived benefits of smoking, are related to behavioral phases of smoking [17-21]. Adolescents who are susceptible to smoking begin to sketch ideas about perception of risks and benefits of smoking. For some, perceived risks and perceived benefits of smoking motivate them either to refuse cigarettes or to experiment [17].

A few studies have observed that perceived long-and short-term physical risks and benefits of smoking associate with different phases of smoking experience among adolescents [22,23]. Although the same studies found that perceived risks of smoking correlated negatively, and perceived benefits of smoking correlated positively, with adolescents’ smoking behavior, they did not compare susceptibility to smoking behavior among nonsmoking adolescents [22,23]. These perceptions of risks and benefits can play an important role in determining the behavior patterns of an adolescent’s susceptibility to smoking and enhance effective intervention and prevention programs [22,24].

In 1983–1984, the first community survey of young people (8–19 years of age) in Nepal was conducted to determine the prevalence of tobacco use among adolescents as well as their attitudes and beliefs about smoking behavior [25]. This study revealed that more than 50% educated adolescents believed (i) smoking is bad for health, (ii) smokers die earlier than non smokers, and (iii) smoking can irritate others. Nonsmoking adolescents also believed that their family members did not want them to smoke [25]. Surprisingly, no other studies have measured such beliefs.

In the United States (US), numerous studies on risk perceptions and benefits of smoking among adolescents and adults have assessed the link between risk and benefit perceptions and tobacco use among adolescents with different smoking experiences [26]. Although such studies are scarce in low-income countries like Nepal, this approach would be highly useful in tailoring and implementing effective tobacco control programs. Therefore, the current study aimed to test the hypotheses that (i) susceptibility to smoking in 14- to 16-year-old adolescents associates with perceived risks and perceived benefits of cigarette smoking, and (ii) perceived risks correlate negatively and benefits correlate positively with susceptibility to smoking.

## Methods

### Study area and population

In October–November 2011, we conducted a population-based cross-sectional study in Jhaukhel and Duwakot Villages in Nepal. Located in the mid-hills of Bhaktapur District, 13 kilometers east of Kathmandu, Jhaukhel and Duwakot represent the prototypical urbanizing villages that surround major urban centers in Nepal, where young people have easy access to tobacco products and are influenced by advertising. Jhaukhel and Duwakot had a total population of 13,669, of which 15% were smokers [27]. Of these, 909 adolescents between 14 and 16 years of age and the male to female ratio was 1.06:1. Among the 909 adolescents, 491 lived in Duwakot [27].

### Sample size

The estimated sample size was based on an unknown prevalence of smoking (50% assumed for conservative sample size estimates), with a required maximum error totaling ± 5% units and a 95% confidence level. Our approach yielded a sample size of 384 adolescents and allowed 20% inflation to account for non-response and incomplete questionnaires. Finally, we decided on a sample size totaling 500 adolescents [28].

Among 500 potential participants, 498 responded, one refused, and one had a hearing impairment. Among the 498 respondents, 485 were nonsmokers and 13 were smokers. Among the 485 nonsmokers, 29.3% were excluded from analysis because they did not answer the questions related to susceptibility to smoking. We performed our final analysis on 352 respondents.

### Study design and sampling method

This was a population-based cross-sectional study. We adopted a proportionate stratified sampling technique to select adolescents from each village. The sampling frame, which included 909 adolescents, was obtained from the baseline survey 2010 [27]. Among the 909 adolescents, 45.9% lived in Jhaukhel and 54.01% lived in Duwakot. Using these sampling fractions, we selected 230 adolescents from Jhaukhel and 270 from Duwakot, yielding 500 randomly selected adolescents. In Step 1, we obtained a sampling fraction that represented 49.8% and 53.2% of the male adolescents from Jhaukhel and Duwakot, respectively (i.e., 114 males from Jhaukhel and 150 males from Duwakot). In Step 2, we further classified the sex of the adolescents into three age groups (14-, 15-, and 16-year-olds) for each village. Among the 114 male respondents in Jhaukhel, 32.2%, 36.1%, and 31.7% belonged to the 14-, 15-, and 16-year-old age groups, respectively. Among 116 female respondents, 42.9%, 28.6% and 28.6% belonged to the same age groups, respectively. In Duwakot, 34.5%, 36.4%, and 29.1% of 150 male respondents belonged to the 14-, 15-, and 16-year-old age groups, respectively, and 30.4%, 38.7% and 30.9% of the 120 female respondents belonged to the same age groups, respectively. Finally, we used systematic sampling from each age group to select the respondents.

### Tools

Our semi-structured questionnaire contained seven major sections: (i) socio demographic and individual information; (ii) smoking activities of family members, relatives, teachers, and friends; (iii) exposure to media and advertising related to tobacco and noncommunicable disease education; (iv) perception of risks and benefits of smoking; (v) smoking behavior of adolescents; (vi) smoking cessation; and (vii) health status. We adapted our questionnaire from the Global Youth Tobacco Survey 2007, the Teen Smoking Question (TSQ), and perceived risks and benefits items from Song et al. and Halpern-Felsher et al. [22,23,29,30]. We modified the original questionnaire to reflect the cultural context of Nepal, and a local public health graduate translated the questionnaire into Nepalese. We made necessary modifications after pretesting the questionnaire in Chagunarayan village, Bhaktapur District. Our study specifically investigated demographic characteristics; perceived physical, social, addiction risks and perceived benefits of smoking; smoking behavior; and susceptibility to smoking.

### Definition of variables

#### Nonsmoker

A nonsmoker adolescent is one who has never smoked, even a puff.

#### Nonsmoker susceptibility to smoking

We determined nonsmoker susceptibility to smoking by asking three questions, using the algorithm of Pierce et al. [14]:

•Will you try a cigarette (taking even just one puff) sometime in the next 6 months? Response choices included (i) definitely will not, (ii) may not, (iii) may be will; and (iv) definitely will.

•If one of your best friends offers you a cigarette, do you smoke? Response choices included (i) never, (ii) sometimes, and (iii) always.

•Do you think you will smoke cigarettes 5 years from now? Response choices included (i) not at all, (ii) slightly likely, (iii) moderately likely, (iv) very likely, and (v) most likely.

Respondents who answered “definitely will not, never, or not at all” to all three questions were considered not susceptible to smoking (code = 0). All other respondents were considered susceptible to smoking (code = 1).

### Perceptions of risks and benefits of smoking

We asked respondents to estimate their likelihood of having various perceptions of the risks and benefits of smoking in a hypothetical scenario: “Imagine that you just began smoking. Sometimes you smoke alone and sometimes you smoke with friends. If you smoke 2–3 cigarettes each day, what is the chance of getting physical and social risks and benefits of smoking?” Respondents estimated the chance (0%–100%) of experiencing seven physical risks (lung cancer, heart disease, facial wrinkles, bad colds, bad cough, bad breath, trouble breathing); two social risks (getting into trouble, smelling like an ashtray); and four perceived benefits (looking cool, feeling relaxed, becoming popular, feeling grown-up) [22,23]. Next, we treated two components (i.e., you can quit smoking cigarettes if you want to, and you will become addicted to cigarettes) as an addiction risk and asked respondents to estimate likelihood (0%–100%), as mentioned above [22].

### Training

Eight local enumerators and two field supervisors attended a 3-daytraining session prior to data collection. The training comprised objectives, ethical issues, ways to build rapport and collect data, questionnaire content, and health hazards of cigarette smoking. At the end of the training session, we pretested the questionnaire in the field and incorporated feedback into the final study questionnaire.

### Data collection

First, enumerators identified households containing adolescents and met with parents to explain the study objectives. After obtaining verbal consent from the parent(s), the enumerators contacted the adolescents and explained the purpose of the study; they also assured the confidentiality of collected information. All adolescents who agreed to participate in the study were interviewed during a 60-minute, face-to-face interview conducted in a separate setting at a time convenient for each respondent.

### Monitoring, supervision and quality control

Field supervisors, a field coordinator, and a PhD student regularly and closely supervised the enumerators. To ensure maximum response rates and reliable data collection, the field supervisors were responsible for spot-checking and discussing field site issues and problems with the field coordinator and the PhD student. In addition, the field coordinator and PhD student randomly cross-checked the completed forms, both in the field and in the office. Erroneous forms were returned to the field for renewed data collection.

### Data management and analysis

Collected data were coded and entered into an EpiData 3.1 program and analyzed using SPSS 17.0 and STATA SE 10 software. Descriptive statistics (i.e., percentage, mean, quartiles, and standard deviation) were computed to describe both categorical and numerical variables (e.g. respondent characteristics and chance estimates for risks and benefits of smoking). Next, we used a chi-square test to compare proportion differences between different categories. Univariate and multiple logistic regressions established the relationship between susceptibility to smoking as a dependent variable and perception of risks and benefits of smoking as independent variables. Before fitting the model, we used principal component extraction with varimax rotation to confirm how well the 13 risks and benefits items loaded on their respective categories. Analysis reduced these 13 items into four meaningful categories, based on the factor scores (factor loading less than 0.04 is not reported). Categories I and II contain items related to perceived likelihood of physical risks, Category III relates to perceived likelihood of the social risks and Category IV relates to perceived likelihood of social benefits [22,23]. Further, physical risks are categorized as physical risk I and physical risk II as bad cold, the short-term risk item, is listed in the first component where all other 3 items(Lung cancer, heart diseases and facial wrinkles) are related with long term risks [23]. Perceived physical risk I includes items describing physical problems caused by habitual smoking (long-term risks and bad cold). Perceived physical risk II comprises items describing short-term risks (bad cough, bad breath, and trouble breathing) of smoking [23]. Perceived social risks included getting into trouble and smelling like an ashtray [22,23]. Perceived social benefits included looking cool, feeling relaxed, becoming popular, and feeling grown-up [23]. Next, we computed the composite scores for the four categories and also for addiction risk. To aid data interpretation and discussion, we coded mean scores into quartiles, where 0 = first quartile and 3 = fourth quartile [23]. Using univariate analysis, we computed the unadjusted odds ratio (OR) according to quartile score for each perception item with susceptibility to smoking. For multiple logistic analysis, we entered all five perception components, including addiction risks simultaneously into the model and computed the adjusted odds ratio (AOR) [23]. We set the significance level at 5% (alpha = 0.05) and excluded missing cases and “do not know” answers.

### Ethical issues

We first sought verbal informed consent from the parents of the participating adolescents as they were less than 18 years of age. Then, we sought verbal informed consent from the participants as well. Further, we informed all respondents that their participation was voluntary and told them they were free to terminate the interview if they did not want to continue or to opt for the next question if they were unwilling to answer a particular question. We also assured all respondents about the confidentiality of collected information. At the end of each interview, we gave the participant a Nepalese-language leaflet that described the harmful effects of tobacco use. Before initiating the study, we discussed the study objectives with local authorities and leaders and obtained their permission. The Nepal Health Research Council and the Ethical Committee of Kathmandu Medical College approved this study.

## Results

### Demographic characteristics

Table 1 explains the demographic characteristics of the 352 participants. The sex ratio was 1.2 males to 1 female, and the mean age of respondents was 14.94 years (SD = 0.81). A majority (97.4%) of respondents were Hindu, followed by Christian (1.4%), Buddhist (0.6%), and other (0.6%). About 83% lived in a nuclear family (father, mother, and children) and all were literate (capable of reading, writing and simple calculations) [31].

**Table 1:** Percentage distribution of demographic characteristics of nonsmoking respondents (2011)

**Items**	**Susceptibility to smoking**	**Non susceptibility to smoking**	**P-value**^**†**^
**Sex**	**n = 175**	**n = 177**	
Male	60.0	48.60	0.03
Female	40.0	51.40	
**Age (years)**			
14	37. 10	33.90	
15	36.0	32.20	0.35
16	26.90	33.90	
**Ethnicity**^**#**^			
Upper caste	50.80	60.50	
Relatively advantaged	42.30	33.30	0.29
Disadvantaged	4.60	3.40	
Dalit (Socioeconomically Disadvantaged)	2.30	2.80	
**Education status (grade)**			
5–10	75.60	73.80	
11–12	24.40	26.20	0.89
**Father’s education**^**‡**^			
Literate^##^	99.50	98.24	
Illiterate	0.50	1.76	
**Mother’s education**			
Literate^##^	78.3	80.70	0.57
Illiterate	21.7	19.30	

^**#**^Ethnic groups are defined according to Nepal Adolescents and Youth Survey, 2011/12 [2]. Upper caste groups (Brahmin, Chhetri, and Thakuri), relatively advantaged group (Newar), indigenous disadvantaged group (Magar and Tamang), and socioeconomically disadvantage (Dalits).

^**†**^Chi-square test applied,^**‡**^ p value cannot be computed because expected value was less than 5.

^##^Literate individuals can read, write, and do simple computation, according to the population monograph of Nepal, 2003 [31].

### Susceptibility to smoking

Among 352 eligible respondents, 49.7% (95% CI: 44.49–54.93) of nonsmokers were susceptible to smoking. The proportion of susceptibility to smoking among males and females differed significantly (P = 0.03). The age wise proportions of susceptibility were not statistically significant (P = 0.35). Similarly, the proportion of susceptibility was not statistically significant for ethnicity, education status, and parental education (Table 1).

### Perception of risks and benefits of smoking: principal component analysis

Table 2 illustrates the findings from factor analysis that used principal component extraction with varimax rotation on 13 items. Perception of risks and benefits of smoking revealed four components with Eigen values >1 (range: 1.17–2.48). Explaining 57.44% of the variation in probability estimates, of which 17.91%, 11.37%, 9.04%, and 19.11% accounted for physical risk I, physical risk II, social risk, and social benefits, respectively.

**Table 2:** Factor analysis using principal component extraction on estimates of perceived risks and perceived benefits of smoking

**Items**	**Rotated factor loading (Varimax)**			
	**Component I**^**#**^	**Component II**^**†**^	**Component III**^**‡**^	**Component IV**^**##**^
Lung cancer	0.64			
Heart disease	0.81			
Facial wrinkles	0.59			
Bad colds	0.55			
Bad cough		0.73		
Bad breath		0.59		
Trouble breathing		0.79		
Getting into trouble			0.85	
Smelling like an ashtray			0.78	
Looking cool				0.77
Feeling relaxed				0.61
Becoming popular				0.83
Feeling grown-up				0.81

Factor loading less than 0.4 is not reported.

^#^Component I: Perception of long term risks of smoking and bad cough (first three items are long term risks of smoking).

^**†**^Component II: Perception of short term risks of smoking.

^**‡**^Component III: Perception of social risks of smoking.

^##^Component IV: Perception of social benefits of smoking.

### Perception of risks and benefits of smoking: descriptive statistics

On average, respondents believed that there was a 70.37% (95% CI: 68.61%–72.14%) chance that physical risk I would occur if they smoked. Similarly, the average chance for physical risk II, addiction risks, social risks and benefits was 86.03% (95% CI: 84.54–87.42); 80.76% (95% CI: 78.65–82.86); 67.57% (95% CI: 65.52–69.64); and 27.08% (95% CI: 25.67–28.45), respectively. For physical risk I, physical risk II, addiction risks, social risks, and social benefits, the interquartile range was 58%–85%, 80%–97%, 70%–98%, 55%–80%, and 19%–35%, respectively.

### Perception of risks and benefits of smoking: logistic regression

Table 3 describes predictors of susceptibility to smoking, based on univariate and multiple logistic regression. Our results showed a non significant positive relationship between perceived physical risk I and susceptibility to smoking (Table 3). The OR of susceptibility to smoking increased 1.21-fold for each increased quartile in perception of physical risk I. In multiple regression, perceptions of physical risk I remained unchanged and non significant (AOR = 1.20). Compared to the first quartile, adolescents in the second, third, and fourth quartile were 2.53-, 2.31-, and 2.06-fold more likely to be susceptible to smoking, respectively.

**Table 3:** Perception as predictor of susceptibility to smoking among Nepalese adolescents, 2011

**Items**	**OR (95% CI)**^**a**^	**AOR (95% CI)**^**b**^
Smoking-related physical risk I	1.21 (0.99–1.47)	1.20 (0.97–1.49)
Smoking-related physical risk II	0.65 (0.53–0.79)	0.63 (0.50–0.77)
Smoking-related addiction	1.48 (1.21–1.80)	1.34 (1.08–1.65)
Smoking-related social risk	0.93 (0.78–1.12)	0.95 (0.77–1.15)
Smoking-related social benefits	1.47 (1.20–1.81)	1.42 (1.14–1.76)

^a^Unadjusted odds ratio (OR) represents a logistic model in which each item was entered separately.

^b^Adjusted odds ratio (AOR) represents a full model included all five independent simultaneously.

OR = Unadjusted Odds Ratio, AOR = Adjusted Odds Ratio, CI = Confidence interval. 95% CI that does not include 1 are significant at P < 0.05. Perceptions were treated as an independent variable and susceptibility to smoking as a dependent variable. The chance estimated (0%–100%) for each items were coded as 0, 1, 2, and 3 for first, second, third, and fourth quartile, respectively. For perceptions of physical risk I, the chance estimates were 10%–58%, 59%–72%; 73%–85%, and 86%–100% for the first, second, third, and fourth quartiles, respectively. For perceptions of physical risk II, the chance estimates were 10%–80, 81%–89%, 90%–97%, and 98%–100% for the first, second, third, and fourth quartiles, respectively. For addiction risk, the chance estimates were 0%–55%, 56%–60%, 60%–80%, and 81%–100% for the first, second, third, and fourth quartiles, respectively. For social risks, the chances estimates were 0%–69%, 70%–89%, 90%–98%, and 98%–100% for the first, second, third, and fourth quartiles, respectively. For social benefits, the chance estimates were 1%–19%, 20%–25%, 26%–35%, and 35%–91% for the first, second, third, and fourth quartiles.

Likewise, susceptibility to smoking was less likely in adolescents who perceived high physical risk II (Table 3). The OR of susceptibility to smoking decreased 0.65-fold (95% CI: 0.53–0.79) for each quartile showing increased perceptions of physical risk II. In multiple regression analysis, the perceptions of Physical Risk II were significant (AOR = 0.63 [95% CI: 0.53–0.79]). Compared to the first quartile, susceptibility to smoking among adolescents in the second, third, and fourth quartile was 0.34-, 0.33-, and 0.27-fold less likely, respectively.

Similarly, perceptions of addiction risk significantly predict susceptibility. The OR of susceptibility to smoking increased 1.47-fold with each increased quartile in perception of addiction risks. When controlling for other perceptions, the AOR of susceptibility increased 1.34-fold with each quartile of increased perception of addiction risk. Compared with the first quartile, the OR of susceptibility to smoking among respondents was 0.56, 1.85, and 2.16 in the second, third, and fourth quartile, respectively.

We determined a non significant inverse relationship between social risks and susceptibility to smoking (Table 3). The OR of susceptibility showed a decreasing trend (0.93-fold) with each quartile of increased perception of social risks; in multiple analysis, the trend was 0.95-fold (Table 3). Adolescents in the second, third, and fourth quartile were 0.78-, 0.95-, and 0.90-fold less likely to be susceptible to smoking compared with first quartile adolescents.

Our results also showed that perceived social benefits of smoking significantly predicted susceptibility to smoking (Table 3). Susceptibility to smoking among adolescents increased 1.47-fold for each increased quartile in perceived benefits of smoking. While controlling for other risks factors, the OR of susceptibility to smoking decreased slightly (1.42-fold). Compared with the first quartile, susceptibility to smoking increased 0.98-, 1.78-, and 2.17-fold for adolescents in the second, third, and fourth quartile, respectively. Similarly, physical risk I, addiction risk, and social benefits correlated positively with susceptibility to smoking (both OR and AOR >1). Physical risk II and social risk correlated negatively with susceptibility to smoking (both OR and AOR <1).

## Discussion

### Findings

This is the first community-based study in Nepal to measure the relationship between perception of risks and benefits of smoking and susceptibility to smoking. Our results reveal several findings regarding susceptibility, perceived risks, and perceived benefits of smoking. This study also provides direction on how to design an effective preventive program to control smoking in adolescents.

We measured perceived risks and perceived benefits of smoking and also tested the hypotheses that (i) susceptibility to smoking in adolescents associates with perceived risks and perceived benefits of cigarette smoking and (ii) perceived risks correlate negatively and perceived benefits correlate positively with susceptibility to smoking. Our findings are consistent with previous studies on the risks and benefits of smoking assessment among adolescents [17,22,23]. To further validate our findings, future studies should test these hypotheses in other settings in Nepal.

Our results provide further empirical support for the contention that adolescents are typically subject to an optimism bias in the process of becoming smokers. This process is consistent with theories that (i) respondents who perceive that smoking does not harm their health and believe in the benefits of smoking show increased susceptibility to smoking; and that (ii) cost-benefit analysis explains both the positives (benefits) and the negatives (risks) of smoking (in relation to susceptibility to smoking) in heath behavior models, including the health belief model, the decisional balance theory, the theory of planned behaviors, and social cognitive theory [17,18,20,21].

The result of our principal component analysis was consistent with previous classification by Harpern-Felsher et al., which described all perceived health consequences as physical risk and all benefits as social and physical [22]. We determined that physical risk I (OR > 1, P = 0.07) and addiction risk (OR >1, P = 0.007) associate positively with susceptibility to smoking. In physical risk I, the OR of susceptibility to smoking decreased with increased quartiles, and overall OR was not statistically significant. These results are consistent with a US study that argued that adolescents generally know the health consequences of smoking but are less aware of its addictive nature [22]. In other words, adolescents might be less concerned about health consequences because they believe that they can quit smoking easily and at any time [22]. Similarly, a report by the US Surgeon General showed that adolescent smokers know the long-term risks of smoking. Thus, knowledge of long-term risks is not a good predictor of smoking behavior [7]. In our study, about 54% of respondents reported 100% certainty that they would be able to quit smoking. Another study showed that 60% of adolescents believe they can smoke for few years and then quit easily [32]. Such data exemplify youthful misconception, indicating that adolescents do not fully comprehend the addictive nature of smoking. In multiple logistic analyses, the likelihood of susceptibility to smoking was 1.42-fold in adolescents who believed in addiction risk compared to non susceptible counterparts. In addition, susceptibility increased with each quartile increase of perceived risk. A Canadian cross-sectional study demonstrated that addiction risk associates strongly with susceptibility to smoking [24]. Thus, addiction risk is an important indicator for susceptibility to smoking [24].

Next, we determined that physical risk II (bad cough, trouble breathing, and bad breath) correlates negatively with susceptibility to smoking (OR < 1, p = 0.000), a finding that concurs with earlier US studies among adolescents at risk of initiating smoking [22]. Additionally, we determined that susceptibility to smoking decreased when belief about physical risk II increased from 0% to 100%. A study by Halpern-Felsher et al. revealed that knowledge about bad cough, trouble breathing, etc., might reduce and prevent smoking among adolescents more powerfully than knowledge of long-term risks [22].

Finally, our results show that perceived social benefits of smoking significantly and positively associate with susceptibility to smoking. Indeed, the OR of susceptibility increased in direct relationship with increased belief of benefits of smoking, from 0% to 100%, concurring with earlier US studies [17,22,23,33]. Another study revealed that adolescents susceptible to smoking perceived fewer negatives and more positives associated with smoking and reported greater temptation to smoke [33].

### Limitations

Because the present study was cross-sectional, we could not infer the causality of the observed association between perceived risks, benefits, and susceptibility. Additionally, since the interview was conducted in respondents’ homes and by local enumerators, information bias might have occurred. Second, due to possible concern about negative social image, we hypothesized that study participants may have underreported their smoking habits, a circumstance that occurs more commonly among females than males [34]. Third, the present study did not include influencing factors related to susceptibility. Finally, we measured perceived risks and benefits only once. These measurements may have affected by the respondents’ characteristics and exposure to anti-smoking campaigns, enumerator motivation, interview site, and the physical conditions of respondents at the time of the interview. It would be interesting to know whether perceived risks and benefits change with time and with other factors, and similarly, how changes in perception would influence smoking behavior.

## Conclusion

We suggest the following important information for a future tobacco intervention program. Perceived short-term physical risks, social risks, and addiction risks and benefits play an important role in susceptibility to smoking among adolescents because such perceptions associate with susceptibility to smoking. Our results suggest that intervention programs for adolescents should focus on the combination of all physical, social, and addiction risks and benefits of smoking. Moreover, establishing an association between perceived risks and perceived benefits of smoking and initiation of smoking among adolescents will require longitudinal studies.

Finally, our results strongly suggest that a successful intervention program to discourage or prevent adolescents from initiating smoking should focus not only on increasing their understanding of long-term physical risks but also draw adolescents’ attention to shorter-term physical risks and actively question their belief that becoming a smoker would make them more socially attractive.

## Competing interests

The authors declare that they have no competing interests.

## Authors’ contributions

URA designed the study, supervised the fieldwork, performed statistical analysis and drafted the manuscript. MP participated in the design of the study, gave statistical advice and supervision and helped draft the manuscript. AK was involved in the design of the study, coordination and supervision and revised the manuscript critically. All authors have read and approved the final manuscript.

## Pre-publication history

The pre-publication history for this paper can be accessed here:

http://www.biomedcentral.com/1471-2458/13/187/prepub
